# Supplementary material for: Pharmacological Inhibition of the Asparaginyl Endopeptidase (AEP) in an Alzheimer’s Disease Model Improves the Survival and Efficacy of Transplanted Neural Stem Cells
Source: Int J Mol Sci. 2023 Apr 23;24(9):7739. doi: 10.3390/ijms24097739 (PMC10178525; doi:10.3390/ijms24097739)
Supplement: Supplementary file 1 [file ijms-24-07739-s001.zip › ijms-2304932-supplementary.pdf]

Supplementary Information

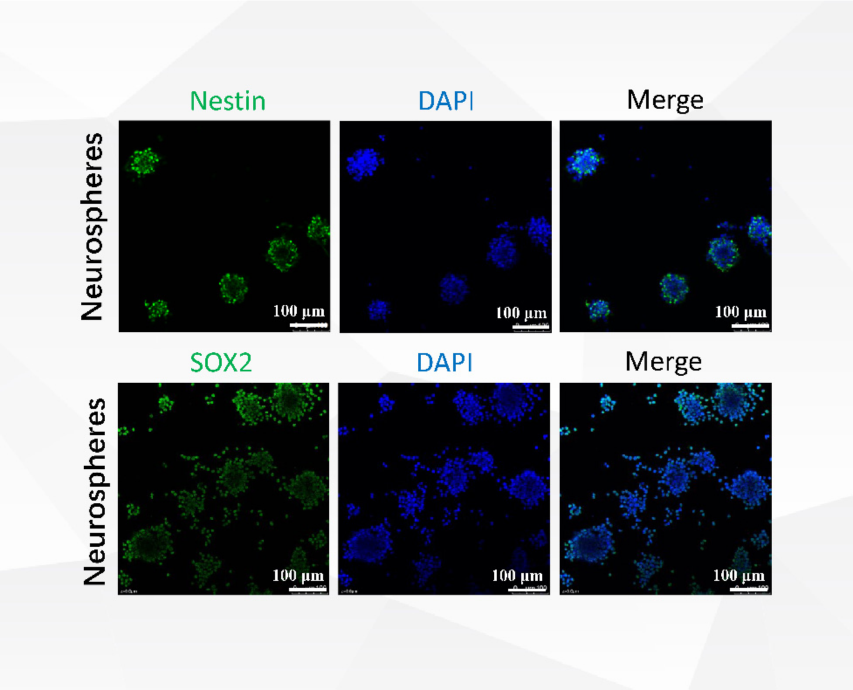

Figure S1. Immunofluorescent staining of neural stem cell marker Nestin and SOX2 in cultured neurospheres.

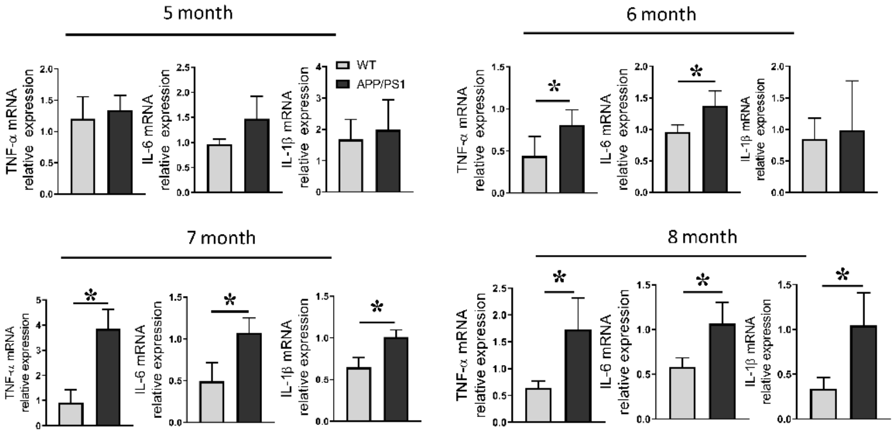

Figure S2. The transcript levels of TNF-α, IL-6 and IL-1β in 5-8 month old WT and APP/PS1 mice brains detected by quantitative real-time RT-PCR. The GAPDH mRNA level was used as an internal reference. *$P < 0.05$, unpaired Student's *t*-test; n = 4 – 5 mice per group.
